# Individualized peer support needs assessment for families with eating disorders

**DOI:** 10.1186/s13030-023-00267-4

**Published:** 2023-03-14

**Authors:** Chisato Ohara, Aya Nishizono-Maher, Atsushi Sekiguchi, Ayako Sugawara, Yuriko Morino, Junko Kawakami, Mari Hotta

**Affiliations:** 1 Department of Clinical Psychology, Faculty of Human Sciences, Bunkyo University, 3337 Minami-Ogishima, Koshigaya-Shi, Saitama, 343-8511 Japan; 2 Department of Behavioral Medicine, National Center of Neurology and Psychiatry, National Institute of Mental Health, Tokyo, Japan; 3 Faculty of Psychology, Meiji Gakuin University, Tokyo, Japan; 4 Narimasu Kosei Hospital, Narimasu Centre for Child and Adolescent Mental Health, Tokyo, Japan; 5 Tokyo Metropolitan Schools, Tokyo, Japan; 6 Atomi University, Faculty of Psychology, Tokyo, Japan

**Keywords:** Eating disorders, Family, Peer support, Caregiver burden

## Abstract

**Background:**

Peer support among family members is important in cases of mental illness, but there has been limited practice or research on individual peer support specific to families taking care of patients with eating disorders (EDs). To conduct peer support activities, it is necessary to clarify the needs of families.

**Objectives:**

The objective of this study are to identify the needs for group and individual peer support and the characteristics of family members with EDs who are willing to receive and provide individual peer support.

**Method:**

A cross-sectional questionnaire survey was conducted for family members with EDs recruited via the Internet. The questionnaires included demographic information on respondents and their patients, questions about the need for family peer support, interest in offering peer support, and social resources. All participants were given the General Health Questionnaire (GHQ-12), the Zarit Caregiver Burden Interview (J-ZBI_8), and the Anorectic Behavior Observation Scale (ABOS).

**Results:**

Out of 314 respondents, 87.3% believed that a group peer support system was necessary, whereas 56.7% believed that an individual peer support system was necessary. As to whether they want to use individual peer support, 70 (22.4%) stated “Extremely YES” and 99 (31.7%) stated “Moderately YES.” Family members who were willing to receive individual peer support used more social resources and had higher scores on the GHQ and J-ZBI_8. Regarding the provision of peer support, 38 (12.2%) responded “very interested and willing to provide it if possible” and 87 (27.9%) responded “interested and willing to study.” Those with a high willingness to provide peer support used more social resources and had lower ABOS scores; however, 38 respondents (45.7%) exceeded the GHQ mental health screening cutoff (3/4).

**Conclusion:**

Family members with ED had a strong need for family peer support Those willing to receive individual peer support suffered from poor mental health and high burden of care. Family members willing to provide peer support tended to have patients whose EDs symptoms had already improved, but their own mental health was not necessarily good. Training for potential peer supporters is needed to implement peer support.

## Background

Eating disorders (EDs) can impose a great burden and distress on families, and the patient and their families may need support. EDs are serious psychiatric disorders characterized by abnormal eating or weight-control behaviors. They often occur at a young age and can easily become chronic [1, 2], and mortality rates are high [3]. ED sufferers often involve their family members in their obsessional thoughts and behaviors about weight, body shape, and food. Families of bulimia nervosa patients also tend to experience financial burdens due to the high cost of food for binge eating and vomiting. Those in charge of the patient’s care (caregivers) carry a particularly heavy mental burden and distress. This burden is often detrimental to the caregivers’ mental health, causing depression or anxiety [4]. A meta-analysis on interventions for caregivers [5] shows that a variety of psychoeducational interventions, including family workshops, self-help materials, and skills-based learning, have reduced career distress. Despite these attempts by professionals, many families have care burdens and deteriorating mental health. Ohara et al. [6] explored the caregiving burdens and mental condition of the primary caregivers for anorexia nervosa patients in Japan. They showed that 60.7% of caregivers indicated a high risk for mental health problems and that affective support from those around the caregiver was an important predictor of mental health. This suggests the need for further expansion of family support.

Peer support among family members or carers with mental illness can be an effective form of family support. In essence, peer support was specifically aimed at enabling the sharing of experience and knowledge with others who are facing similar issues and to provide social and emotional support [7]. There are two main types of peer support settings: group peer support and individual peer support. Group peer support includes self-help groups, internet support groups, and peer-led or peer-run psychoeducation programs. Group peer support plays a major role in family support for severe mental illnesses such as schizophrenia. Under community-based family-to-family (F2F) support [8, 9], professionally trained family peer supporters provide group psychoeducation programs for families with schizophrenia and other severe mental disorders. A systematic review showed that the caregivers who participated in the F2F support programs reported a significant decrease in their burden and an increase in social support and family function [10]. However, individual peer support consists of mentor arrangements, whereby a “novice” carer is matched with a more experienced carer. This is sometimes referred to as “parent-to-parent consultation” or “peer-to-peer support” [11]. Individual peer support is often targeted at care providers with physical illnesses such as asthma, diabetes, juvenile rheumatoid arthritis, and chronic illnesses [7, 12]. A randomized controlled trial has shown that a peer-to-peer support program for parents with emotionally disturbed children leads to positive program effects, including increased perceived benefit of engagement, more engagement in their child’s services, and a more positive response to social norms [13]. The stated aims of intervention are to provide informational, affirmational, and emotional support [14].

The provision of individual peer support may be a useful option for supporting families, but it is unclear what peer support needs families with EDs have. Rhodes et al. showed that adding parent-to-parent consultations early in the Maudsley model of family-based treatment for anorexia may have therapeutic effects [15], and the qualitative analysis of that study shows that the individual peer support provided was an intense emotional experience that helped family members to feel less alone, feel empowered to progress, and reflect on changes in family interactions [16]. While these studies suggest the potential effectiveness of individual peer support, it is not clear to what extent peer support is needed in the community. Regarding the need for group peer support, a survey of family associations for people with EDs in Japan identified a need and group peer support practices in this area, such as self-help groups. Various existing group interventions may also incorporate peer support among family members [5]. However, studies on individual peer support are limited, and it is unclear to what extent peer support for EDs is needed. Furthermore, there is a lack of clear information on families willing to provide individual peer support. This study will focus on identifying the needs and characteristics of those seeking and willing to provide individualized peer support in families with EDs.

The objectives of this study are to identify the following:

- 1. the need for group and individual peer support,
- 2. the characteristics of family members who are willing to receive individual peer support, and.
- 3. the characteristics of family members willing to provide individual peer support.

## Method

### Subjects

The inclusion criteria for this study were being over 20 years of age and having a patient with ED in their family. The diagnostic criteria were briefly explained, and the patient’s diagnosis of an ED was self-determined by the participants; a physician’s diagnosis was not required. The potential participants were recruited through the website of the Japan Association of Eating Disorders. We have done publicity for associations of families with EDs throughout Japan, as well as for several medical institutions.

### Procedure

A cross-sectional questionnaire survey was conducted. The data collection period was between November 1, 2020 and February 10, 2021. The explanatory documents, consent forms, and questionnaires were distributed to those who offered to participate, either by mail or by hand. Six hundred copies of the questionnaire were distributed. Consent forms and completed questionnaires were collected by mail. Each respondent was paid a reward worth 1,000 yen (about $9.00 at the time).

### Assessment

The questionnaire consisted of the following:

1. demographic information on respondents and their family member patients

The respondents were evaluated using demographic information, including their relationship with the patient, their age, whether they were the primary caregiver or not, and whether they were living together with the patient or not. We also collected background information on the patient, including their age, height, weight, their lowest past weight at the current height, diagnostic subtypes of EDs, history of hospitalization, history of the use of emergency medical services, and comorbid mental disorders. The patient’s Body Mass Index (BMI) was calculated based on their height and weight.

2) Need for family peer support

We defined family peer support, noting that “individual peer support” refers to one-on-one face-to-face counseling, and the following questions were asked:

- 1. Do you think individualized family peer support is necessary?
- 2. Do you think family peer support group is necessary?
- 3. Would you like to have individualized family peer support?
- 4. Would you like to have family peer support group?

Each item is evaluated on a five-point scale.

3) Interest in studying or offering peer support

We asked about interest in studying or offering peer support, using a four-point scale: “Very interested and would like to offer if possible,” “Interested and willing to study,” “interested but don’ t have enough time.,” and “not interested.”

4) Social resources

We asked if they had used any of the following social resources:

1. Discussion with the patient’s physician,
2. Dialog with professionals other than the attending physician (e.g., psychologists, nutritionists, nurses, etc.) who were involved with the patient,
3. Dialog among family members with EDs,
4. Counseling and family therapy,
5. Lectures by specialists,
6. Lectures by parties (patients and recovering patients) and family members,
7. Ongoing participation in family meetings and family classes,
8. Books/DVDs/TV programs/Internet (websites, SNS, videos, etc.) by experts, and
9. Books/DVDs/TV programs/Internet (websites, SNS, videos, etc.) by parties/families.
10. The Japanese version of the General health questionnaire (GHQ-12)

The GHQ-12 is a popular screening scale used to measure general psychological health in a variety of settings [17]. We used the Japanese version [18]. It assesses symptoms of anxiety, social dysfunction, self-doubt, and depression. The GHQ-12 is a 12-item, 4-point scale using a “past two weeks” time frame designed to assess and detect psychiatric morbidity, with higher scores indicating poorer health. The GHQ-12 is rated on a 4-point Likert scale and uses two scoring methods. Using the Likert method, “not at all” = 0, “no more than usual” = 1, “rather more than usual” = 2 and “much more than usual” = 3. Using the GHQ method, “not at all” = 0, “no more than usual” = 0, “rather more than usual” = 1, and “much more than usual” = 1. The 3/4 cutoff point of the GHQ method score was used for screening for a possible mental disorder, and the Likert method score was used for other statistical analyses.

6) Japanese version of the Zarit caregiver burden interview (J-ZBI_8)

The Zarit Caregiver Burden Interview assesses the burden on caregivers [19]. The eight-item short Japanese version (J-ZBI_8) has a high level of reliability and validity, comparable to that of the full version [20]. Each item was evaluated on a 5-point Likert scale from 0 (Never) to 4 (Nearly always). A higher score indicates a higher caregiving burden.

7) Anorectic behavior observation scale (ABOS)

The ABOS is a 30-item questionnaire that evaluates the patient’s ED and cognitive problems based on their family’s observations [21]. The Japanese version has been validated [22]. Each item is related to the patient’s condition over the previous month. A response is scored as 2 if the problem is certainly present, 1 if the problem has not been seen or its existence is uncertain, and as 0 if the problem is certainly not present. A higher score indicates more severe symptoms from the family’s perspective.

8) General functioning subscale of the McMaster family assessment device (GF-FAD)

The FAD is based on the McMaster Model of Family Functioning and has been widely used in the field of mental illness [23]. The Japanese version has been validated [24]. The General Functioning Subscale of the FAD (GF-FAD) consists of 12 items scored from 1 to 4. A higher score indicates that the respondent sees the family’s functioning as poorer.

### Statistics

Wilcoxon’s signed rank test was conducted to compare the need for individual peer support with the need for group peer support. To identify the characteristics of those with high peer support needs and those with low peer support needs, the group was divided into three groups according to peer support needs, and a one-way analysis of variance (ANOVA) and chi-square analysis of three factors were conducted. Similarly, a one-way ANOVA and chi-square analysis of four factors were conducted on the willingness to provide peer support. Subjects with missing values of approximately one fourth or more of the total were excluded from the analysis of this study. Meanwhile, for those with fewer missing values, items from each missing score were excluded from each analysis according to the pair-wise deletion method.

## Results

Responses were obtained from 325 respondents (for a response rate of 54.2%). Of these, 314 were included in the analysis.

### Clinical and demographic data of responses

The demographics of the respondents and their patients are shown in Table 1.

**Table 1:** Clinical and demographic Information

		N	%	Mean	SD
Respondent variables					
Relationship	Mother	234	74.5%		
	Father	34	10.8%		
	Others	46	14.6%		
Age	20–29	12	3.8%		
	30–39	19	6.1%		
	40–49	64	20.4%		
	50–59	131	41.7%		
	60–69	66	21.0%		
	70≦	22	7.0%		
Primary caregiver	Yes	239	76.4%		
	NO	74	23.6%		
Living with patient	Yes	220	70.3%		
	NO	93	29.7%		
Total GHQ				4.6	3.8
Total ZBI_8				12.3	7.9
Total ABOS				22.2	11.3
Total GF-FED				26.1	6.7
Patient variables					
Age	< 10	1	0.3%		
	10–19	69	22.0%		
	20–29	127	40.6%		
	30–39	78	24.9%		
	40–49	29	9.3%		
	50≦	9	2.9%		
Age of onset				17.2	5.9
BMI				16.8	3.6
Lowest BMI				13.4	2.6
Type of eating disorder or condition	AN-R	112	40.1%		
	AN-BP	67	24.0%		
	BN	42	15.1%		
	BED	35	12.5%		
	Duplication of disease type	2	0.7%		
	Almost recovered	21	7.5%		
Social status	In education	108	34.7%		
	Working	141	45.3%		
	Neither working nor in education	62	19.9%		
History of hospitalization	Yes	207	67.0%	3.0	3.3
	No	102	33.0%		
Use of emergency medical services	Yes	83	26.7%		
	No	228	73.3%		
Psychiatric comorbidities	Yes	179	57.0%		
	No	135	43.0%		

Of the 325 respondents, 234 (74.5%) were mothers; 34 (10.8%) were fathers; 18 (5.7%) were partners; and 28 (9.0%) fell into the category of “others.” In terms of age, 12 (3.8%) were in their 20 s; 19 (6.1%) in their 30 s; 64 (20.4%) in their 40 s; 131 (41.7%) in their 50 s; 66 (21.0%) in their 60 s; and 22 (7.0%) in their 70 s or older. Of the respondents, 239 (76.4%) were primary caregivers, and 220 (70.3%) lived with the patient; 157 (51.6%) exceeded a GHQ cutoff of 4 or more points, and 177 (63.7%) rated patient symptoms above the ABOS cutoff of 19 or more.

Regarding the patients’ characteristics, 303 (96.5%) were female and 10 (3.2%) were male; one patient in care (0.3%) was less than 10 years old; 69 (22.0%) were in their teens; 127 (40.6%) were in their 20 s; 78 (24.9%) were in their 30 s; 29 (9.3%) were in their 40 s; 6 (1.9%) were in their 50 s; and 3 (1.0%) were in their 60 s or older. The mean age of onset was 17.2 ± 5.9 years. Regarding current medical condition, 21 (7.5%) had almost recovered; 112 (40.1%) had AN-R; 67 (24.0%) had AN-BP; 42 (15.1%) had BN; and 35 (12.5%) had BED. In terms of which medical condition had lasted the longest, AN-R was the most common condition with 112 patients (40.1%). The mean current BMI was 16.8 ± 3.6 and 13.4 ± 2.6 for those patients who had the thinnest body types. The social status of the patients was as follows: 108 (34.4%) were in education; 141 (44.9%) were working; and 62 (19.7%) were neither working nor in education. Regarding the history of hospitalization, 207 (67.0%) had a history of hospitalization, with an average of 3.0 ± 3.3 hospitalizations. Eighty-three (26.7%) had a history of emergency medical visits. One or more comorbidities were present in 179 patients (59%): depressive disorders in 80 (25.4%), neurodevelopmental disorders in 40 (12.7%), bipolar disorder in 25 (8.0%), panic disorder in 23 (7.3%), borderline personality disorder in 23 (7.3%), alcohol use disorder in 12 (3.8), post-traumatic stress disorder in 6, and other disorders in 19 (0.6%).

### Peer support needs and willingness to provide

**Fig. 1:**
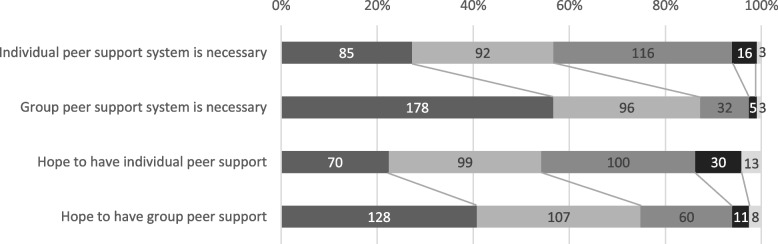
Need for individual and group family peer support. The bars show the number of respondents out of 314 participants who answered as follows,  Extremely necessary,  Moderately necessary,  Undecided,  Not very necessary,  Not necessary

The need for peer support is shown in Fig. 1. Regarding the social need for one-on-one face-to-face consultation by family peer supporters (individual peer support), 85 respondents (27.1%) answered “Extremely necessary” and 92 (29.5%) answered “Moderately necessary,” whereas 116 (36.9%) answered “undecided,” 16 (5.1%) answered “not very necessary,” and 3 (1.0%) answered “not necessary.” Similarly, when asked if they would like to receive one-on-one face-to-face counseling themselves, 70 (22.4%) said “Extremely YES,” 99 (31.7%) said “Moderately YES,” 100 (32.1%) said “undecided,” 30 (9.6%) said “Moderately NO,” and 13 (4.2%) said “NO” (4.2%). Wilcoxon’s signed rank test was conducted to compare the need for individual peer support with the need for group peer support, and the results showed that social need (Z =  − 9.609, *p* < 0.001, *r* = 0.54) and hope to receive (Z =  − 8.120, *p* < 0.001, *r* = 0.46) were both significantly different and that there was greater need for group peer support than for individual peer support.

**Fig. 2:**
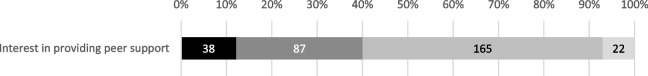
Interest in providing individual family peer support. The bars show the number of respondents, out of 314 participants, who answered as follows

Regarding interest in providing peer support (Fig. 2), 38 (12.2%) were “very interested and willing to provide it if possible,” 87 (27.9%) were “interested and willing to study,” 165 (52.9%) were “interested but don’ t have enough time.,” and 22 (7.1%) were “not interested.”

### Relation between the need for individual family peer support and other factors

The association between the need for peer support (three factors: extremely = Extremely YES, moderately = Moderately YES, NO = Neither and Moderately NO and NO) and respondent and patient factors were examined in the ANOVA and chi-square test. The results of the ANOVA (Table 2) showed significance in the effects of the number of resources used (F(2, 309) = 6.041, *p* < 0.01), total J-ZBI_8 score (F(2, 303) = 3.176, *p* < 0.05), and total GHQ score (F(2, 300) = 3.273, *p* < 0.01); multiple comparisons using Tukey’s b test showed that in both cases, there was a significant difference between “very much want to receive” and “undecided or do not want to receive.” In other words, the “very willing” group had used more resources and had higher total J-ZBI_8 and total GHQ scores than the “undecided or unwilling” group. The results of the chi-square test showed a significant difference in the need for peer support depending on the relationship with the patient (χ2(4) = 10.444, *p* = 0.034), and the residual analysis showed that mothers were more likely than fathers and other family members to say they were “very willing to receive” and less likely to say they were “undecided or unwilling.” Similarly, there was a significant difference depending on whether the respondent was a primary caregiver or not (χ2(2) = 7.117, *p* = 0.028), with primary caregivers receiving fewer “undecided or unwilling” responses than non–primary caregivers. No significant differences were found for other factors: patient age, diagnosis, history of hospitalization, history of emergency care visits, presence of psychiatric comorbidities, age of respondent, and whether or not the respondent lived with the patient.

**Table 2:** Analysis of variance between each factor and need for peer support

	N	YES		Moderately YES		NO		*p*	Turkey's b test’ for clarity
		average	SD	average	SD	average	SD		
Number of social resources used	312	6.1	2.3	5.6	2.4	4.9	2.7	**	YES > NO
Age of onset (y)	306	17.1	5.4	17.7	7.0	17.0	5.3		
Current BMI (kg/m2)	275	16.6	3.4	16.3	3.6	17.2	3.6		
Lowest BMI (kg/m2)	282	13.5	2.4	13.2	2.8	13.5	2.6		
Number of hospitalizations	204	2.8	2.8	2.6	2.8	3.3	3.8		
GHQ	302	17.9	6.7	16.8	6.1	15.5	6.6	*	YES > NO
J-ZBI_8	306	14.4	7.4	12.4	7.5	11.5	8.1	*	YES > NO
ABOS	276	22.0	9.9	23.6	10.6	21.4	12.4		
GF-FAD	296	25.7	6.8	26.1	5.8	26.2	7.3		

YES = "Extremely necessary", Moderately YES = "Moderately necessary", NO = "undecided," "not necessary," or "not necessary", *GHQ* Total score of General Health Questionnaire 12, *ZBI_8* Total score of the Japanese version of the Zarit Caregiver Burden Interview, *ABOS* Total score of the Anorectic Behavior Observation Scale, *GF-FED* General Functioning Subscale of the McMaster Family Assessment

^*^p < .05, ** *p* < .01

### Associations between willingness to provide peer support and other factors

The association between willingness to provide peer support and respondent and patient factors was examined in the ANOVA and chi-square test.

The results of the ANOVA test (Table 3) showed significant effects in the number of resources used (F(3, 308) = 9.192, *p* < 0.001), age of onset (F(3, 302) = 3.573, *p* < 0.05) and total ABOS score (F(3, 272) = 6.705, *p* < 0.001). According to multiple comparisons using Tukey’s b test, the number of resources used was higher in the “very interested and willing to offer if possible,” “interested and willing to try to study,” and “interested but don’t haveenough time.” groups than in the “not interested” group. Age at onset was lower in the “interested and willing to study” group than in the “not interested” group, and total ABOS scores were lower in the “very interested and willing to study” group than in the “interested and willing to study,” “interested but don’t have enough time” and “not interested” groups. The mean of the total score on the GHQ in the “very interested and willing to offer if possible” group was 7.1 ± 1.2, which was not different from the other groups; 16 out of 38 (45.7%) respondents exceeded the cutoff (3/4) for possible mental health issues.

**Table 3:** Analysis of variance between each factor and willingness to provide peer support

	N	Willing to provide (A)		Willing to study (B)		Cannot afford (C)		Not interested (D)		*p*	Turkey's b test' for clarity
		Average	SD	Average	SD	Average	SD	Average	SD		
Number of social resources used	312	6.2	2.1	5.7	2.4	5.3	2.6	3.0	2.6	***	A, B, C > D
Age of onset (y)	306	16.8	7.0	15.9	4.1	17.9	5.8	22.6	11.9	*	B < D
Current BMI (kg/m2)	273	18.1	4.2	16.8	3.3	16.5	3.6	17.0	3.2		
Lowest BMI (kg/m2)	281	13.9	2.6	13.4	2.6	13.2	2.7	14.5	2.6		
Number of hospitalizations	204	2.5	3.1	2.3	1.9	3.5	3.9	2.6	0.6		
GHQ	302	7.1	1.2	6.3	0.7	6.5	0.5	6.5	1.4		
J-ZBI_8	306	12.8	8.3	11.6	7.8	12.3	7.9	7.4	1.6		
ABOS	276	15.5	9.4	22.6	11.9	22.9	10.9	29.4	10.1	***	A < B, C, D
GF-FAD	296	26.3	7.0	24.8	6.3	26.5	6.8	6.8	1.5		

A = "Very interested and would like to offer if possible", B = "Interested and willing to study", C = "Interested, but no capacity for training", D = "Not interested", *GHQ* Total score of General Health Questionnaire 12, *ZBI_8* Total score of the Japanese version of the Zarit Caregiver Burden Interview, *ABOS* Total score of the Anorectic Behavior Observation Scale, *GF-FED* General Functioning Subscale of the McMaster Family Assessment

^*^p < 0.05, ***p < 0.001

In the chi-square test, there was a significant difference in the relationship with the patient (χ2(6) = 24.034, *p* < 0.001), and the residual analysis showed that the “not interested” response was significantly less frequent among mothers than among the other groups. Fathers were significantly less likely to respond, “very interested and would provide, if possible,” and mothers were significantly more likely to respond “very interested and would provide if possible.” There was a significant difference in whether the respondent was a primary caregiver or not (χ2(3) = 15.567, *p* = 0.001), and the residual analysis showed that primary caregivers were more likely to respond, “very interested and would like to provide if possible” and less likely to respond “not interested” than those who were not primary caregivers. There was also a significant difference in the history of emergency care visits (χ2(3) = 9.060, *p* = 0.028), with those who had a history of emergency care visits being less “not interested” than those who did not. No significant differences were found for the other factors.

## Discussion

This study revealed the acute need for group peer support among family members of patients with EDs. More than half of such family members feel the need for individual peer support as well. Family members willing to receive individual peer support suffer from poor mental health and high care burden, and they make heavy use of existing social resources. Cases where family members were willing to provide individual peer support accounted for 12.2% of the respondents. The following is a detailed discussion.

### Need for group peer support and need for individual peer support

The activities of Japanese associations for the families of those with EDs shaped our conclusion that group peer support was more necessary than individual peer support among these families. While group peer support is already available for those with EDs, individual peer support is not. Japan has about 50 family associations for those with EDs, and these associations are considered to provide peer support to family member [25]. However, Japan presently lacks a one-to-one peer support system, and the lack of familiarity with and popularity of such a system may have contributed to this situation. Although this study focused primarily on individual peer support, group peer support, especially peer-led family psychoeducation, plays a major role in family support for mental illness, and its effectiveness has been verified [10]. Such needs may be acute in cases of EDs as well, and further research and program development may be needed in the future.

### Percentage and characteristics of those who need individual peer support

As for one-on-one individual peer support, 70 (22.4%) said “Extremely YES,” and 99 (31.7%) said “Moderately YES,” indicating that more than half of them would like to receive such support. However, the rest did not actively want to receive peer support, and it should be noted that not all families need it. Family members with a significant need for peer support had higher scores for care burden (J-ZBI 8) and mental health (GHQ). However, there was no difference in the patients’ BMI or in the severity of symptoms from the family’s perspective (ABOS). Therefore, it is not the severity of the patient’s symptoms but rather the deterioration of the care provider’s own mental health and the high burden of care that lead to an increase in the need for peer support. This should be considered when providing peer support, and consultants should provide assistance to reduce the burden of care and ease their minds. Primary caregiver status and being the mother of a patient were also associated with a significant need for peer support. In previous studies, mothers were overwhelmingly the primary caregivers, probably because the burden of care was higher for primary caregivers. In addition, families who were willing to receive peer support were more likely to have used social resources such as medical care, counseling, family associations, and support groups, which were correlated with the level of caregiver burden rather than with patient severity. Some studies have noted that peer support is not a substitute for professional treatment but supplementary to it [16, 26]. Therefore, family members with a significant need for peer support and who are already using a variety of resources may not be satisfied with those resources and want additional peer support.

### Percentage and characteristics of those willing to provide peer support

As for willingness for providing peer support, 38 (12.2%), 87 (27.9%), and 165 (52.9%) answered “very interested and willing to provide it if possible,” “interested and willing to study,” and “interested but don’t have enough time, respectively. Many participants were interested in providing peer support, but only some of them were willing to provide it.

Family members willing to provide peer support tended to have patients whose ED symptoms had already improved. Characteristic of the respondents willing to provide peer support were their low ABOS scores, referring to the current severity of illness from the family’s point of view. There was no difference in the lowest BMI, history of hospitalization, or history of emergency care visits. Therefore, patients whose family members were willing to provide peer support do not differ from other groups in terms of past disease severity but their symptoms of EDs have already improved. This is consistent with the concept of one-on-one peer support, in which family members in cases of advanced recovery provide support to newcomer family members. However, there was no difference in mental health between families willing to provide peer support and those unwilling to do so. Sixteen out of 38 (45.7%) respondents were willing to provide peer support, which exceeds the cutoff for possible mental health issues. Those who provide peer support may not necessarily have high levels of good mental health and still feel the burden of providing care. Therefore, a system in which professionals educate and support potential peer supporters is considered necessary for the safe provision of peer support. As with the need for peer support, the primary caregivers, who are mothers, were more likely to respond that they would like to provide peer support. Conversely, those who responded that they were not interested tended to care for patients with a high age of onset (22.6 ± 11.9), in addition to the fact that they were not mothers and had used few social resources. These family members may have had less experience in making a commitment to the patient’s care, which may have led to their indifference to providing peer support. Those who responded that they were not interested in peer support were less likely to use existing social resources. One reason may be that their gratitude and trust in the social support they have received may have increased their interest in providing peer support. Alternatively, it is possible that individuals whose personalities are more sociable or who prefer to consult with others are more interested in social resources, such as peer support.

### Limitations and strengths of this study and future issues

First, a limitation of this study is the possible bias because of the use of a web survey. People who were able to access the Internet and were interested in receiving or providing peer support may have been more likely to respond to this survey. Therefore, these results may not be directly applicable to all families who take care of EDs. Second, the diagnosis of an ED provided by the participants may not have been accurate. Third, this survey was conducted only among residents of Japan, thus it may have been influenced by cultural factors. Although individual peer support for families of cancer patients and peer support as group psychoeducation for schizophrenia exist, the concept of peer support for families itself does not seem to be very widespread in Japan. It is possible that potential needs are not being picked up due to a lack of concrete images of peer support.

However, the strength of this survey is that the results were obtained from a diverse population, including families of patients who do not have access to medical institutions.

Our future research should seek ways to develop specific peer support programs based on the the peer support needs of the family, as identified in this study. To establish individualized peer support systems in communities, it is necessary to support and educate potential peer supporters. This study found that those interested in peer support tend to already have multiple social support systems and that even those willing to provide peer support are not necessarily in good mental health. Therefore, additional social support systems are needed, and it is conceivable that support and training by experts will be necessary to cultivate peer supporters. We are developing a training program for families who have expressed interest in providing peer support. We are also planning to offer one-on-one peer consultation. In the future, it will be necessary to validate the process of training peer supporters and to verify the effectiveness of their consultation.

## Conclusions

This study revealed a high need for peer support among family members of patients with EDs. While there is a greater need for group peer support, it was found that there is also some need for individual peer support. Further, this study identified the characteristics of those who would be interested in receiving and providing individual peer support. Both those who wanted to receive and provide peer support used more social support than those who were not interested in peer support. Family members willing to receive individual peer support suffered from poor mental health and a high burden of care. Family members willing to provide peer support tended to have patients whose symptoms of EDs had already improved, but their own mental health was not necessarily good. Training for potential peer supporters is needed before a peer support program is implemented.

## Data Availability

Not applicable.
